# Methods for Recovering Microorganisms from Solid Surfaces Used in the Food Industry: A Review of the Literature

**DOI:** 10.3390/ijerph10116169

**Published:** 2013-11-14

**Authors:** Rached Ismaïl, Florence Aviat, Valérie Michel, Isabelle Le Bayon, Perrine Gay-Perret, Magdalena Kutnik, Michel Fédérighi

**Affiliations:** 1 LUNAM Université, Oniris, UMR1014 SECALIM, Nantes, F-44307, France; E-Mails: rached.ismail@oniris-nantes.fr (R.I.); michel.federighi@oniris-nantes.fr (M.F.); 2 INRA, UMR1014 SECALIM, Nantes, F-44307, France; 3 ACTALIA, Produits laitiers, La Roche-sur-Foron, F-74801, France; E-Mails: v.michel@actalia.eu (V.M.); p.gay-perret@actalia.eu (P.G.-P.); 4 FCBA, Bordeaux, F-33028, France; E-Mails: Isabelle.LEBAYON@fcba.fr (I.L.B.); Magdalena.KUTNIK@fcba.fr (M.K.)

**Keywords:** wood, packaging, food contact, microorganisms, recovery methods, contamination

## Abstract

Various types of surfaces are used today in the food industry, such as plastic, stainless steel, glass, and wood. These surfaces are subject to contamination by microorganisms responsible for the cross-contamination of food by contact with working surfaces. The HACCP-based processes are now widely used for the control of microbial hazards to prevent food safety issues. This preventive approach has resulted in the use of microbiological analyses of surfaces as one of the tools to control the hygiene of products. A method of recovering microorganisms from different solid surfaces is necessary as a means of health prevention. No regulation exists for surface microbial contamination, but food companies tend to establish technical specifications to add value to their products and limit contamination risks. The aim of this review is to present the most frequently used methods: swabbing, friction or scrubbing, printing, rinsing or immersion, sonication and scraping or grinding and describe their advantages and drawbacks. The choice of the recovery method has to be suitable for the type and size of the surface tested for microbiological analysis. Today, quick and cheap methods have to be standardized and especially easy to perform in the field.

## 1. Introduction

Various types of surface such as plastic, stainless steel, glass and wood are used today in the food industry. These surfaces are subject to contamination by microorganisms, some of which are able to form biofilms. Contamination of surfaces depends on their characteristics, such as smooth, rough, porous, or irregular, and their state, for example before or after the cleaning process, new or old, dry or wet. Moreover, in the food-processing industry, the contamination of surfaces by microorganisms is sometimes required, such as the surface of wooden shelves used in ripening cheese [1], or for making vinegar [2]. Nevertheless, in both cases, deliberate or undesirable contamination, a method of recovering microorganisms from the different solid surfaces is necessary as a tool for protecting consumer health. 

As described below, the contamination of surfaces can be a public health problem, and in fact some disease outbreaks were subsequently found to be due to surface contamination. For example, Gill *et al.* showed that inadequately cleaned equipment contributed to the contamination of meat by *Escherichia coli* and the unambiguous role of the equipment surface in contact with meat [3]. Allen *et al.* described poultry contamination by equipment in the slaughterhouse when poultry meat from broiler flocks, otherwise negative for *Campylobacter*, might have been contaminated if the previously slaughtered flock had been positive and the bacteria had remained on the equipment surfaces [4].

Although outbreaks of *Escherichia coli* O157:H7 have been linked to consumption of contaminated ground beef, the organism is rarely isolated from the implicated meat. In 1993 in California, five family members who ate hamburgers from meat purchased from a local market and cooked “medium rare”, reported diarrhea [5]. The elderly woman died three weeks after hospitalization. Different samples of ground beef, obtained from three local markets, were tested and five were positive, identifying violations in grinding procedures of one market. Another outbreak due to *E. coli* O157:H7 on a supermarket meat grinder was described by Banatvala *et al.* [6]. This study demonstrated that the grinder was responsible for contamination because this equipment was cleaned only once a week and not disinfected. *E. coli* O157 has been identified as the source of outbreaks with fresh produce. Rangel *et al.* showed that 21% of *E. coli* O157:H7 outbreaks from 1982 to 2002 in the United States were due to fresh produce [7]. Beuchat *et al.* described different fresh produce vectors of *E. coli* O157 outbreaks such as apple cider, lettuce, radish, alfalfa sprouts and other mixed salads [8]. The ability of *E. coli* O157 to survive on fresh produce surfaces could be responsible for cross-contamination on cutting boards. In 2008, Arthur *et al.* studied contamination of cattle and swine slaughterhouse lairage [9]. They described the persistence of *Salmonella* and *E. coli* O157:H7 in flooring materials being responsible for meat contamination. 

A *Salmonella* study carried out by Berends *et al.* on pork at cutting plants and at the retail level described contamination of surfaces [10,11]. They showed that contaminated carcasses were constantly being brought into cutting lines. Even if surfaces and utensils were cleaned and disinfected during breaks and at the end of the working day, this would most likely prevent not more than about 10% of all cross-contamination that takes place during a working day.

Working surfaces are also responsible for cross-contamination of products by contact with *Listeria monocytogenes*. McLauchlin described *L. monocytogenes* as bacteria able to survive in environments where there is moist organic material, like food-processing surfaces [12]. Moreover, *L. monocytogenes* has the ability to grow on a wide variety of foods at refrigeration temperatures and to resist common preserving techniques. It is a particular concern as a contaminant during processing. Actually, Vogel *et al.* showed that *L. monocytogenes* survived desiccation for three months in a simulated food processing environment [13]. Wenger *et al.* analyzed production line product samples [14] and found 0 to 8% of samples from the stages before the peeler-conveyor belt apparatus were *L. monocytogenes*-positive whereas 12 out of 14 (86%) samples collected from this apparatus were *L. monocytogenes*-positive. These data confirmed cross-contamination of products after contact with contaminated equipment. Peccio *et al.* studied two plants and detected *L. monocytogenes* in equipment and tools, occasionally at a very low level [15]. Kneaders and mincers, used in the pig meat processing facility, and knives, used in the beef slaughterhouse, were contaminated by *L. monocytogenes* even after cleaning procedures. However, it was not found in fridge rooms or floor drains. 

The HACCP-based process is now widely used for the control of microbial hazards to prevent food safety issues. According to this preventive method, the use of microbiological analyses of surfaces has appeared as one of the tools to check good hygienic practices and to maintain a high level of hygienic production of foods. In Europe, food contact is regulated by ECR 852−2004 [16]. This regulation specifies that measures should be taken to ensure safe contact between products and packaging material and to avoid chemical contamination. There are specific standards for pathogens such as *Listeria monocytogenes* [17,18].

To date, methods and techniques have been published and are well-known for bacteria and fungi contained in soil or in rock [19]. The review of Hirsch discussed methods for the study of rock deterioration, for example, by microorganisms. These included non-destructive methods for the study of monuments and more destructive methods to apply when rock samples were collected. In the food industry, methods and techniques to recover microorganisms from surfaces have been developed, but parameters such as the diversity of experimental conditions or samples hinder the choice of the best method [20]. For any one method, a variety of results may be obtained; for example different recovery rates. In an optimum method, the tool, the reagents and the equipment must produce the same end result even if they are used by different operators under the same sampling conditions. The review of Giudici *et al.* [21] covered the technological and microbiological aspects of traditional balsamic vinegar. These authors demonstrated that liquid-foodstuff needed an extraction method to recover microorganisms from the liquid surface. In fact, methods were developed to identify and quantify acetic acid bacteria growing on the surface of vinegar. This heterogeneous group of strictly aerobic bacteria was recognized as “vinegar bacteria” because of their role in the bioconversion of ethanol into acetic acid [2]. Since 1958, it has been well-known that a recovery method is suitable for a certain kind of surface and inappropriate for another food contact surface [22]. However, recovery methods for smooth surfaces are mostly agreed upon although there is no real consensus for an acceptable standardized method [23]. Standardized recovery methods for microorganisms on rough surfaces, such as wood which have specific porosity, are still lacking. The international standard ISO 18593:2004 presents two techniques currently used for smooth surfaces [24], but does not specify methods for scraped plastic or porous material like wood for instance.

The aim of this review is to present the most frequently used methods in order to provide a basis to evaluate the microbial load on common surfaces used in cheese, fruit, vegetable and meat processing *i.e.*, mainly stainless steel, plastic and wood. In this review, according to the poor literature devoted to this subject, we choose to use the term “recovery” to describe the method for extracting microorganisms from different surfaces for microbiological analysis. First, methods are described which can be considered “non-destructive” because they do not damage the surfaces before microbiological analysis. Secondly, “destructive” methods that could damage surfaces of interest are reviewed. Before the conclusion, a table summarizing these methods is presented to highlight their advantages and drawbacks, their practicality in processing plants, the rate of recovery, surface type, and sample size.

## 2. Non-Destructive Recovery Methods

### 2.1. Swabbing Methods

As conventional swabbing is the recommended method [24], it is commonly used on plastic, stainless steel [25] and wood [26] and practically applied in field studies or food safety management protocols in industry to detect pathogenic bacteria. 

Conventional swabbing procedures use a sterile cotton swab with an applicator stick for releasing microorganisms from surfaces [27]. The cotton swab bud applied on a material surface recovers bacteria and fungi spores and releases them into the extracting solution during a vortexing step, followed by direct plating or dilution plating [28]. Some authors proceed by rubbing on agar plates of a selective or non-selective growth medium. The recovery rate of this standard swabbing method was described by Davidson *et al.* [29]. In the conventional way, a classic hygienic cotton swab is employed dry or (if the tested surface is dry) often moistened in 0.1% sterile peptone water [25], in Maximum Recovery Diluent (MRD) [29] or another extracting buffer solution if a detergent or a disinfectant used on the surface must be neutralized. Then, the swab is immersed briefly in the initial buffer solution or directly in a nutrient broth [23,25]. Finally, the microorganisms are enumerated according to the current regulations. 

An unconventional swab can be used like a calcium alginate swab bud which, unlike a cotton swab bud, dissolves directly in the culture medium [30], meaning that the microorganisms delivery is probably easier than with traditional cotton swab buds*.* Studies published in the fifties and sixties compare recovery rates of different types of swabs. For example, according to Higgins [31], the use of calcium alginate wool as a substitute for non-absorbent cotton-wool for swabs to be used in quantitative work seemed to be justified, since the recovery of organisms was much greater. In 1985, one study compared different swab (cotton and aluminum; cotton and plastic; calcium-alginate and aluminum and Dacron^®^ and plastic) to isolate *Chlamydia trachomatis* from clinical specimen [32]. The recovery of *C. trachomatis* varied markedly depending upon the type of swab. Two calcium alginate swabs were toxic to cell cultures, resulting in no visible inclusions. The remaining calcium alginate swab yielded a 77% bacterium recovery. Two of three Dacron^®^-on-plastic swabs were toxic to the cells, and the third yielded a recovery of 81%. The single cotton-on-wood swab tested appeared to be inhibitory to *C. trachomatis*. Another unconventional swabbing method consists in soaking a classic sterile cotton swab in a chemiluminescent buffer before sampling the surface [23]. Instead of quantifying contamination indicators with classic microbiological solid or liquid, selective or non-selective media, this method indicates the presence of these bacteria by detecting beta-galactosidase using a luminometer. 

Thus, ATP bioluminescence-based analysis has been widely adopted during recent decades for risk assessment and monitoring surface cleanliness. However, this method has the disadvantage of detecting organic debris too, which explains the disparity of the results reported in the literature [27]. It should therefore not be used for quantitative studies since the results cannot be correlated to surface area (cm^2^ or piece). Moreover, some studies showed similar data between luminescence and microbiological methods whereas others demonstrated significant differences [23]. Apart from these limitations, ATP bioluminescence provides a rapid response when monitoring cleaning procedures, which enables immediate remedial action during an industrial process.

Hodges *et al.* described a macro foam swab protocol to recover *Bacillus* spores from a stainless steel surface and showed a recovery of up to 49.1% of spores from 10 cm^2^ steel surfaces [33]. Asséré *et al.* showed that swabbing did not detach all bacteria cells [34]. For example, on a conveyor belt material, of the *Pseudomonas fluorescens* cells that survived chlorine treatment, only 2% were recovered by swabbing.

### 2.2. Friction (Scrubbing) Methods

For sampling large surfaces (>100 cm^2^), the standard ISO 18593:2004 recommends the use of sterile sponges or fabrics instead of swabs [24]. Some authors reported that cellulose ones were at least as effective as traditional cotton swabs with a better ability to be moistened and absorb fluids [35]. In fact, this kind of scrubbing tool might allow a higher pressure application on the tested surface. As the whole surface could be tested, these techniques might allow access to a real-time image of the microbial load. Mariani *et al.* described another technique of vigorous scrubbing using a toothbrush followed by a step of rubbing with wipes [1]. This method was compared with sonication and showed similar results for both techniques. This scrubbing technique is currently used in artisanal and industrial cheese dairies to recover microorganisms when contamination is suspected.

Although swabbing methods are considered efficient for detecting microorganisms on any surface [36], several authors pointed out limitations that should be taken into account when using them. For example, poor reproducibility between operators has been reported and explained by different pressures applied on the surface [37]. Regarding recovery rates, Carpentier [20] and Moore *et al.* [23] highlighted that only a small proportion of the total bacterial population present on the surface could be recovered, particularly from a dry surface. This low recovery capacity could be linked to the fact that the collected microorganisms seem to remain trapped in the tip of the swab, instead of being easily released. A vortexing or shaking step is therefore unavoidable in order to help recover the microorganisms from the swab or sponge. Brown *et al.* described an evaluation of a surface sample method using polyester-rayon blend wipes for collecting *Bacillus* spores from non-porous materials such as stainless steel by a sonication extraction method [38]. This study showed that the efficiency of recovery by wiping was more related to the texture than the porosity of the surface. This work also demonstrated that the detection limit of this method may imply that negative wipe samples did not guarantee the absence of microorganisms on the surface. 

Despite these limitations, swabbing or scrubbing procedures are still more appropriate methods for removing microorganisms from difficult-to-clean or irregular surfaces than printing methods [23]. Moreover, swabbing methods are suitable for detecting various germs when contamination is suspected to be heterogeneous or to come from different microorganisms in the same sample. Yet, these techniques cannot assess the inner layers in which microorganisms can lie because of the specific porosity of some surfaces such as wood. 

### 2.3. Printing Methods

This is the second method along with swabbing which is recommended by standard ISO 18593:2004 for verifying the efficacy of hygiene procedures for slaughterhouse materials [24]. The quantification and viability analysis of bacteria on solid surfaces can be evaluated by agar contact methods. The aim of this technique is to carry out a printing of microorganisms present on the solid surface. It has the advantage of being used without preparation of the sample and may only need an incubation phase. However, the tested surface should be clean enough to give an acceptable result with not too many colonies. 

Among the most common methods, agar plates can be cited. First described by Hall and Hartnett in 1964, this consists of pressing contact plates of selective or non-selective medium onto the solid surface tested and then incubating the contact plates under appropriate conditions [39]. Disinfection and washing procedures could damage microorganisms and lead to an increase in the incubating time to 14 days otherwise the counting would be underestimated. Usually, 55 or 65 mm diameter contact plates are stamped onto five different parts of a large surface sample. Cervenka *et al.* described a “coupon-printing method” which was tested only in the laboratory. Stainless steel, polypropylene and glass 1.5 × 1.5 cm chips were put on a selective agar medium for 5 minutes then aseptically recovered in order to incubate the plates and observe the viable cells [40]. The results were used to compare the persistence of bacteria on three surfaces and it was concluded that *Arcobacter butzleri* was more persistent on plastic. However, no quantitative information could be drawn from these results. In fact, printing methods are often chosen in studies concerning large environmental surfaces in hospitals and in foodstuff companies as an alternative to the swabbing method. Yet, it is interesting to note that only a small proportion of the microorganisms on the surface are recovered. Khamisse *et al.* showed that this technique recovered only 1% of a population enumerated in parallel by a smear method after 14 days of incubation [41]. To determine the total aerobic microbial contamination, there is another agar contact method using commercial dipslides. This test consists of ready-made dipslides with agars suitable for detecting microorganisms of interest on opposite sides of the slide. The dipslides are firmly pressed for a few seconds against the surface tested before being incubated according to the supplier’s recommendations. The imprints are evaluated by counting the colonies. Because of a lack of accessibility to the microorganisms lying in crevices and breaches, Moore *et al.* [23] showed that this method was not suitable for irregular surfaces or porous materials or for highly contaminated environments, previously described by Niskanen *et al.* [42], while Peneau *et al.* added it was not suitable for food-contact surfaces [43]. The reproducibility of the printing method can be improved by using a constant application force (with a weight or a spring). Appropriate devices are available on the market.

There is also a microorganism recovery standard which is intended as a basic screening test for hygienic equipment design. It consists of pouring the agar directly onto the surfaces as described by the working group EHEDG “Test method” to evaluate procedures for cleaning equipment [44] and is not a routine microbiological method used in the food industry. In the laboratory, cleaning a piece of industrial machine is compared to a straight piece of pipe. The cleanability is assessed by bacterial counts before and after the cleaning, with a low detergent, of test pieces previously in contact with a ‘soured milk soil’ containing bacteria. 

## 3. Destructive Recovery Methods

### 3.1. Rinsing and Immersion Methods

Often used to detect and enumerate bacteria present within food processing or packaging environments, the rinsing method exists in multiple variations within different studies. In general, the surface of interest (chips or surface patches) or sampling tool (swab, sponge) is placed in a sterile plastic bag with a filter. The weight of the surface sample is calculated and sterile buffer is added (for example: 1% sodium citrate, distilled water). Then, the plastic bag is shaken according to a specific protocol: more or less vigorously for a short or a long time. This step could be destructive for the surface sample. At the end, the rinsing solution is collected under sterile conditions for microbiological analysis. 

Ak *et al.* tested different soaking methods to recover microorganisms present on plastic and wooden boards [45]. Typically, the tested surface remained around one minute in contact with the solution before the rinse was diluted and spread over non-selective or selective agar media. Obviously, and according to one study about the efficacy of several microbiological methods for turkey carcass enumeration [46], the more microorganisms were heterogeneously spread on a surface, the more effective the rinsing method was. These results were in contrast with those of localized methods such as swabbing and scrubbing. However, this method is less effective than destructive methods, especially for porous, irregular materials, in which microorganisms could penetrate. Moreover, rinsing methods can only be applied to removable pieces of material which have a limited size. 

### 3.2. Sonication Methods

Sonication is the use of ultrasound to break cellular membranes or molecular aggregates, in order to clean or disinfect the surface by using an ultrasonic-bath. Some medical publications deal with the monitoring of microorganisms on solid surfaces for hygienic and health issues [47,48]. The use of ultrasound (sonication) to dislodge biofilms from the surface of implants that have been removed can increase the detection limit of microbiological studies [49]. Sonication is currently used, coupled to immersion and agitation methods, to recover pathogenic bacteria biofilms from solid surfaces which are often made of metal or stainless steel. 

Mettler *et al.* used this method to study flooring material in the agri-food industry and the relationship between hygienic quality and surface texture [50]. Stainless steel plates of 10 cm^2^ and tiles, washed according to cleaning procedures, were tested and the residual contamination was recovered by immersion in a neutralizing solution coupled to sonication (4 min, 28 kHz). The results showed that the mean roughness of a material is not a proof of cleanability and hygienic quality. In 2008 Cervenka *et al.* used a similar method on plastic and stainless steel to study the persistence of *Arcobacter butzleri* on these two surfaces (25 cm²) [40]. In this study, sonication was applied at 38 kHz for 5 minutes and the results proved that *Arcobacter* could survive for a long time on a surface with a low surface free energy like plastic compared to a higher surface free energy like metal. Mariani *et al.* in 2007 used a sonication method on wooden cheese shelves in order to study the ecology of the biofilm in contact with cheeses [1]. The sonication was carried out by using a portable sonicator providing a resonant frequency of 40 kHz for 10 seconds directly on 10 cm^2^ samples. Then, suspended biofilm was collected in tubes containing Ringer’s solution.

Le Bayon *et al.* described in 2010 a technique coupling vacuum pressure and sonication to recover bacteria and fungi spores from wood surfaces and showed higher yields with this method than with grinding [51]. These results could be explained by the fact that ultrasound allows microorganisms to be recovered from the wood matrix without leading to significant mortality. 

Sonication is an interesting method when coupled with immersion, scrubbing or vacuum pressure for example, but it seems to be difficult to apply outside of the laboratory and tends to give similar results to other more convenient methods. For example, Rose *et al.* concluded that ultrasound was as efficient as the swabbing method for recovering *Bacillus* spores from stainless steel [52]. An alternative method could be the one described by Kang *et al.* where quantitative recovery methods for *Listeria monocytogenes* applied to stainless steel surfaces were evaluated [53]. These authors compared swabbing, rinsing, printing and sonication with a sonicating brush head method, which consisted of scrubbing followed by sonication (280 Hz, 10 s). For the stainless steel samples tested, this technique led to the highest recovery level.

### 3.3. Scraping and Grinding Procedures

Destructive methods give the best recovery rates for surface materials which are irregular or porous. Microorganism penetration in this kind of surface seems to concern the first 2–3 mm of the surface according to a study reported by Mariani *et al.* about wooden shelves used in ripening cheese [1]. Because of their hardness, it seems impossible or difficult to apply these methods to stainless steel or plastic surfaces. 

Grinding can rarely be used as a recovery method in industry for two reasons: firstly, it is destructive for the sample and secondly, the sample should be thin to be ground directly, situations seldom encountered in the field. Indeed, chopping boards or wooden shelves used in ripening cheese have a thickness ranging from 1.5 cm to 5 cm. Finally, in some publications, grinding refers more to a surface sample preparation step than to the recovery method itself as described in the study of Vainio-Kaila *et al.* [54]. Grinding was done earlier but no more details were provided. Moreover, the scraping or planing off process refers to physical cleaning procedures [55], for example raking wooden or plastic boards in cheese-ageing cellars.

The table below (Table 1) summarizes the various techniques of microorganism recovery from surfaces used in the food-processing industry. The data are taken from the references cited in the text. 

**Table 1:** Microorganism recovery methods from surfaces used in the food-processing industry.

Method	Destructive	Applicable surface type	Sample size	Advantage	Drawback
**Swabbing**	No	Smooth	Small (Localized)	* Standardized * Practicality in processing plant * Variable contamination ^(a)^	* Dependent on operator * Poor reproducibility * Low recovery rate
**Friction**	No	Smooth or rough	Large >100 cm²	* Practicality in processing plant	* Dependent on operator * Poor reproducibility * Low recovery rate
**Printing**	No	Smooth	Large >100 cm²	* Standardized * Practicality in processing plant * Variable contamination ^(a)^	* Dependent on operator * Poor reproducibility * Low recovery rate
**Rinsing, immersion**	Yes	All	Small, large	* Practicality in processing plant * Variable contamination ^(a)^	
**Sonication**	Yes	All	Small, large	* Practicality in processing plant * In-depth extraction	
**Scraping, grinding**	Yes	All	Small	* In-depth extraction	

^(a)^ Variable contamination indicates greater or lesser concentrations of a microorganism on the same surface.

## 4. Conclusions

The choice of the recovery method has to be appropriate for the study of the microorganism and the working surface. In fact, there are so-called “alternative” molecular methods, developed in order to extract genomic DNA to identify the microbial composition of foods such as vinegar [56] and follow its manufacturing process. However, these molecular methods do not provide an immediate answer to companies about the presence of living microorganisms potentially harmful to the health of the consumer. Thus, to control microbiological hazards in the food industry, the recovery method has to be suitable for the type of surface studied (accessible, smooth, porous) and the sample size required for microbiological analysis (Table 1). Today, it could be assumed that simple printing or scrubbing procedures are more suitable for plastic or stainless steel surfaces as microorganisms could be more easily recovered from smooth material as described in *Listeria monocytogenes* biofilm studies [57,58]. However, Midelet *et al.* showed that polyvinyl chloride (PVC) could be an irregular surface and could offer microorganisms shelter to survive [59]. Because of its porosity and its desiccation properties, wood needs its own extraction method(s). Actually, a porous surface could offer hiding places in which microorganisms could survive and maybe even form a biofilm whereas desiccation could lead to the death of microorganisms. Indeed, swabbing and contact methods did not give good results with this material [60]. Other methods to detect and quantify microorganisms on wood have already been developed but require specific equipment [61,62]. This review has highlighted the difficulty of comparing different methods of recovering microorganisms from surfaces because many parameters can change from one study to another, for example the “stage” of strains (during the growth phase or not). Indeed, Carpentier and Cerf [63] noted that Chasseignaux *et al.* [64] showed that the favorable conditions for *L. monocytogenes* presence in the field were all unfavorable conditions for *L. monocytogenes* adhesion but favorable for *L. monocytogenes* growth [65]. Another parameter that can change the recovery rate is indicated by the dependent experimental conditions of each study. In fact, the fraction of microorganisms detached from surfaces with a particular recovery method may vary and therefore result in different values of the recovery rate. The calculation of this rate is also important. For example, Midelet *et al*. [59] carried out successive swabbings on a given area of surface. The bacteria enumerated for these successive swabbings enabled Veulemans method [66] to be used to estimate the total number of recoverable bacteria on a given area of surface. In fact, Veulemans equation accounts for the slope of the recovery curve established thanks to successive swabbings. This study showed that using this equation gave a better assessment of the bacterial load on a polymer surface than only one swabbing did. A recovery method has to be standardized in laboratory conditions and then tested in the field. However, a method with a high recovery rate in the laboratory could be unsuitable for wild-strain detection by classic microbiological analysis. In addition, bacteria have a propensity to form biofilms in real conditions, such as on the surfaces of food industry equipment [67]. Several studies have indicated that various bacteria, including *Escherichia coli*, *Staphylococcus aureus and Salmonella spp.*, survive on hands, sponges/cloths, utensils and currency for hours or days after initial contact with the microorganisms [68,69,70]. Kusumaningrum *et al.* [70] showed that the presence of residual foods and the level of contamination on stainless steel surfaces may have an important role as it may improve the survival of *Salmonella enteritidis, Staphylococcus aureus* and *Campylobacter jejuni* for several hours or even days.

Haeghebaert *et al.* suggested that 40.5% of all food-borne infection outbreaks registered in 1998 in France were linked to contamination by equipment with biofilms [71]. Biofilm formation is a well-known bacterial mode of growth and survival on food surfaces as Reuter *et al.* described for *Campylobacter jejuni* in the food chain and during transfer between hosts [72]. Barnes *et al.* suggested that surface roughness may play an important role in the adhesion of microorganisms by protecting them from shear forces and increasing the available surface area [73]. In this study, greater numbers of *S. aureus* adhered to untreated steel (with the rougher surface). In the same study, the authors showed cross-contamination linked to raw and processed foods by food contact surfaces such as stainless steel.

Today, quick and cheap methods have to be defined and standardized which are especially easy to perform in the field. Innovative methods are needed to better control microbiological hazardous events on equipment and working surfaces in the food industry, especially for porous and irregular material. 
